# Dose-Dependent Effects and Metabolomic Analysis of Foliar-Applied Carbon Dots on Cotton (*Gossypium hirsutum* L.) Growth

**DOI:** 10.3390/plants15172659

**Published:** 2026-08-30

**Authors:** Qiong Wu, Wen Cao, Yudong Chen, Yuehan Liu, Eryang Li, Guanghui Lv

**Affiliations:** 1 College of Ecology and Environment, Xinjiang University, Urumqi 830046, China; 2 Key Laboratory of Oasis Ecology of Education Ministry, Xinjiang University, Urumqi 830046, China

**Keywords:** cotton, carbon dots, physiological responses, metabolomics, growth promotion

## Abstract

While carbon dots (CDs) are promising green nanomaterials for sustainable agriculture, how they regulate cotton growth via physiological and metabolic reprogramming remains poorly understood. This study evaluated foliar CD application (0–200 mg·L^−1^) to uncover dose–response patterns and metabolic drivers. CDs exerted a concentration-dependent impact, with 100 mg·L^−1^ yielding optimal results. This application markedly boosted shoot and root biomass (fresh weight: 103.43% and 44.28%; dry weight: 56.03% and 40.39%) and enhanced specific leaf area by 52.51% over the control. CDs improved photosynthetic efficiency (elevated Pn, Gs, and E values, alongside increased chlorophyll *a* and carotenoids) and strengthened antioxidant defenses (enhanced leaf POD/SOD and root POD/CAT activities). Despite mild MDA increases indicating oxidative stress, accumulated proline and soluble sugars in roots suggested adaptive osmotic adjustment. These findings suggest that CDs maintain physiological homeostasis by modulating antioxidant defense and osmotic adjustment. Untargeted metabolomics identified 2440 metabolites. Phenylpropanoid biosynthesis and flavonoid biosynthesis were the most prominently enriched pathways based on KEGG enrichment results. K-means and correlation analyses revealed key metabolites linked to cotton biomass. Overall, CDs facilitate cotton development through synergistic physiological and metabolic reprogramming, underscoring their potential as innovative agricultural growth regulators.

## 1. Introduction

As a primary global fiber and economic crop, cotton (*Gossypium hirsutum* L.) plays a pivotal role in international agriculture. Its productivity and fiber characteristics are directly related to the stable development of textile industry and agricultural economy [1,2]. Because cotton growth is readily affected by multiple factors, and given the multiple challenges posed by limited land resources, climatic stress, and pests and diseases, improving cotton growth, achieving yield increase and stability, and enhancing stress tolerance have become central objectives of modern plant protection and cultivation research [3]. As a burgeoning field, nanobiotechnology applications have shown great potential in plant nutrition, disease control, photosynthetic regulation, and stress tolerance [4]. Driven by their unique optical, charge, and surface-modifiable features, carbon dots (CDs) have emerged as a focal point within this field [5].

Typically scaling below 10 nm in diameter, CDs are identified as zero-dimensional (0D), quasi-spherical carbonaceous nanostructures characterized by inherent fluorescence [6,7]. Owing to the robust photostability, good biocompatibility, and facile surface functionalization, they have been widely applied in sensing, photocatalysis, and bioimaging [8]. In addition, CDs exhibit extraordinary promise in the agricultural field, attracting widespread attention [9,10]. In plant science, it has been reported that CDs are assimilated and translocated following foliar or rhizosphere application [11,12]. These nanomaterials regulate key metabolic activities, such as seed germination, root morphogenesis, photosynthetic efficiency, and enzymatic antioxidant profiles, ultimately promoting plant development and improving tolerance to abiotic challenges [13]. However, different synthesis methods and surface functional groups confer distinct biological effects on CDs, and their concentration-dependent effects exhibit biphasic responses, ranging from promotion to inhibition, in various plant materials. This highlights the importance of systematically evaluating the dose and application mode of CDs [14].

Environmental conditions dictate the steady-state levels and structural diversity of metabolites, which represent the ultimate signaling outputs of metabolic networks. Metabolomics systematically characterizes changes in metabolic profiles and links plant growth phenotypes to environmental responses, making it an important approach for elucidating plant–environment interaction mechanisms [15]. Multi-omics insights reveal that CDs can act as novel plant biostimulants to promote crop growth. For instance, carbon quantum dots (CQDs) applied via foliage can remodel carbohydrate metabolism and amino acid biosynthesis pathways in melon (*Cucumis melo* L.), thereby improving fruit sweetness and quality [16]. Similarly, the application of nitrogen-doped CDs (N-CDs) can modulate molecular networks to optimize metabolism, reinforce the antioxidant system, and promote cell wall development, thereby bolstering stress tolerance in rice seedlings [17].

Overall, as an emerging plant bioregulator, CDs have shown the potential to promote growth, improve agronomic traits, and enhance defensive capacity across diverse crop species. However, their effects are contingent upon material source, surface properties, and application concentration. In particular, it remains unclear how different CD concentrations regulate cotton development and physiological traits, and which metabolic biomarkers and pathways are involved in these responses. Therefore, cotton was utilized for the present investigation, and foliar spraying was performed with CDs at six concentration levels. We systematically assessed the impact of varying CD concentrations regarding cotton development, focusing on physiological traits and metabolic profiles. By integrating metabolomics with pathway analysis, this study further elucidated the molecular mechanisms underlying cotton growth regulation and identified key metabolic biomarkers and pathways potentially involved in the response to CDs. The findings serve to lay the scientific groundwork for integrating CDs into sustainable cotton cultivation, while concurrently providing insights into the development and utilization of nanobiotechnology for crop regulation.

## 2. Results

### 2.1. Characterization of CDs

**Figure 1:**
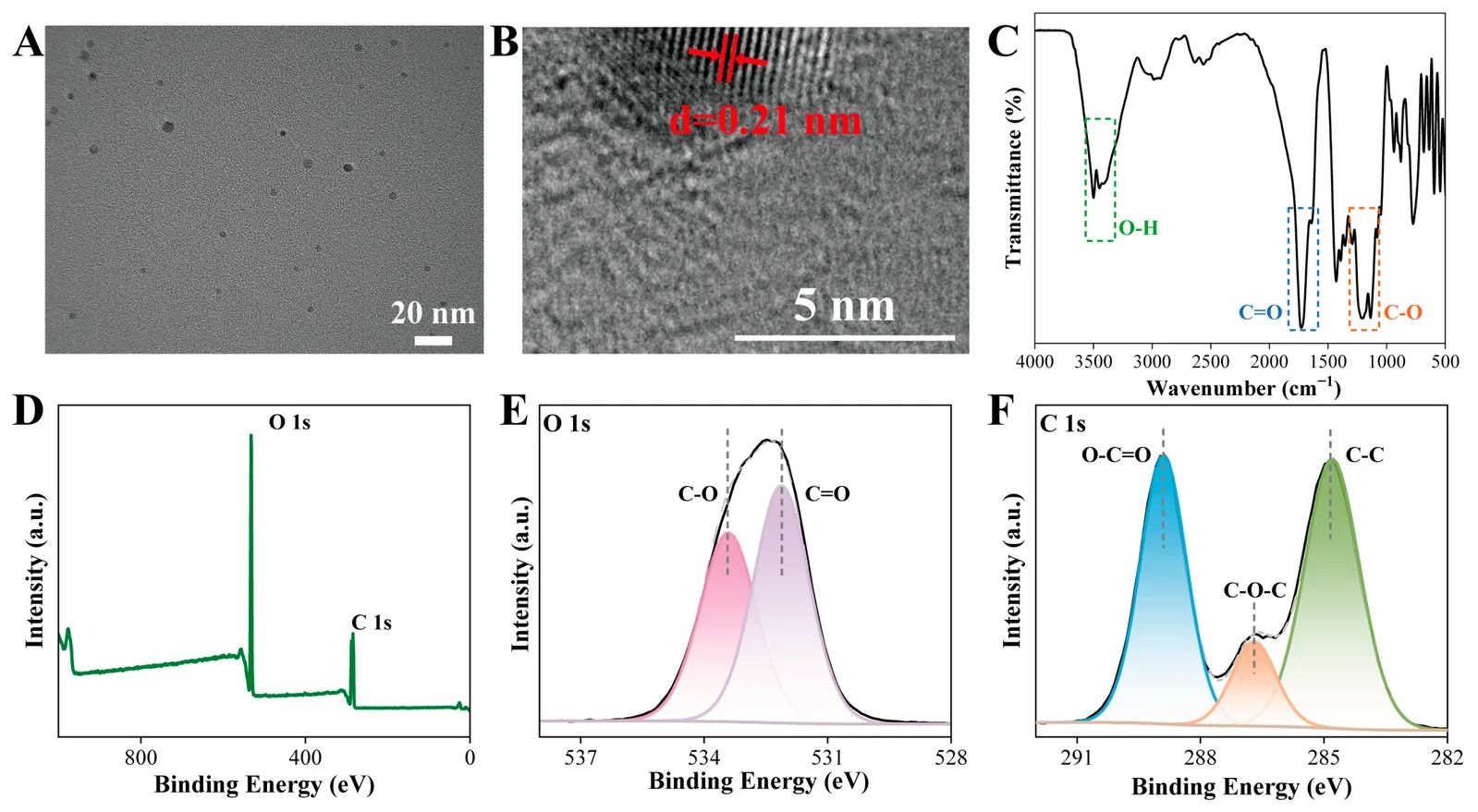
Characterization of CDs. (**A**) TEM images of CDs. (**B**) HRTEM images of CDs. (**C**) FTIR spectrum of CDs. (**D**) Full XPS spectrum of CDs. (**E**) The high-resolution XPS spectra of O 1s, and (**F**) C 1s for CDs.

Morphology characterization via TEM exhibited spherical nanostructures for the CDs with a homogeneous size range (Figure 1A). HRTEM images further confirmed the excellent crystallinity of the CDs, as evidenced by well-defined lattice fringes corresponding to an interplanar spacing of roughly 0.21 nm (Figure 1B) [18]. The CDs were uniform and monodispersed, with an average particle size of 3.64 ± 1.08 nm calculated from the size distribution histogram (Figure S1). In terms of chemical composition and structure, the XPS survey spectrum (Figure 1D) showed that carbon and oxygen are the primary constituents of the CDs. Furthermore, the presence of C=O and C-O bonds was validated by the O 1s region (Figure 1E), featuring binding energy peaks centered at 532.08 eV and 533.38 eV, respectively, thereby elucidating the surface chemical states of oxygen. Signals identified in the C 1s high-resolution region (Figure 1F) at 284.78, 286.68, and 288.88 eV were attributed to C-C, C-O-C, and O-C=O linkages, respectively [19]. Such FTIR results were highly consistent with the XPS analysis: the characteristic absorptions at 1211 cm^−1^ (C-O), 1429 cm^−1^ (C-H), and 1727 cm^−1^ (C=O), together with a prominent O-H stretching region (3000–3500 cm^−1^), collectively confirmed that the CDs surface was rich in hydrophilic functional groups (Figure 1C) [20]. The distribution of these functional groups suggests that the CDs possess excellent hydrophilicity and structural stability, enabling broad applications in the field of biology.

### 2.2. Cotton Growth and Physiological Responses Under CDs Exposure

**Figure 2:**
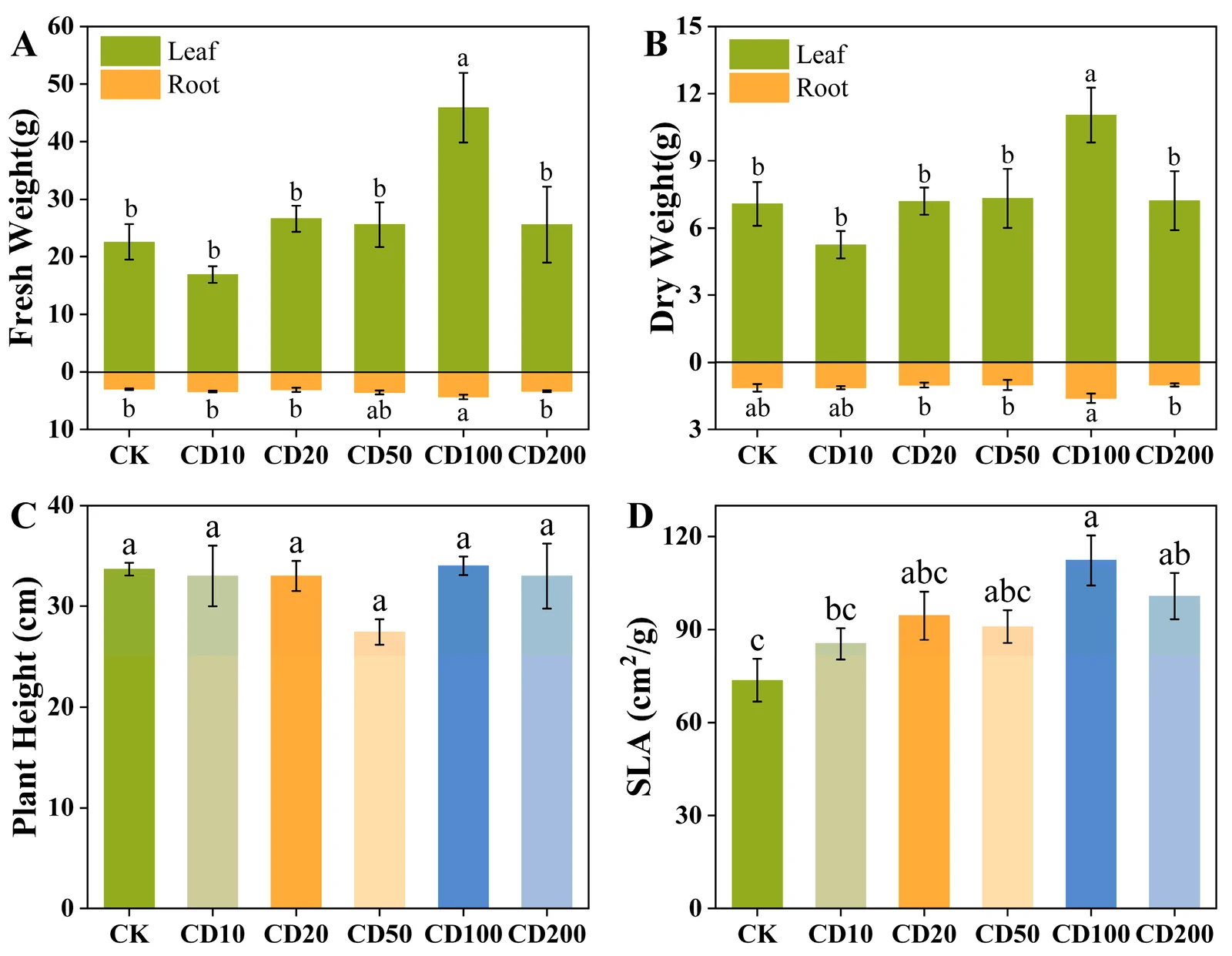
Effects of different concentrations of CDs on cotton growth parameters. (**A**) Fresh weight; (**B**) dry weight; (**C**) plant height; (**D**) specific leaf area (SLA). Error bars represent the mean ± standard error (*n* = 4), and lowercase letters indicate significant differences at the *p* < 0.05 level.

When the CDs concentration gradient was set at 0–200 mg·L^−1^, the growth parameters of cotton exhibited a clear dose-dependent response. As shown in Figure 2, after CDs treatment, the dry and fresh weights and specific leaf area of cotton first rose and subsequently decreased, whereas plant height showed no obvious difference among the groups. Under 100 mg·L^−1^ CDs treatment, all morphological indicators of cotton reached their maximum values. Relative to the control, substantial increments were observed in both fresh weights (103.43% for leaves and 44.28% for roots; Figure 2A) and dry weights (56.03% for leaves and 40.39% for roots; Figure 2B). Additionally, the specific leaf area increased by 52.51% (Figure 2D). Although plant height reached its maximum under 100 mg·L^−1^ CDs exposure, the variations across all groups lacked statistical significance (Figure 2C). This indicates that plant development can be stimulated by CDs at certain concentrations, exhibiting a dose–response relationship.

**Figure 3:**
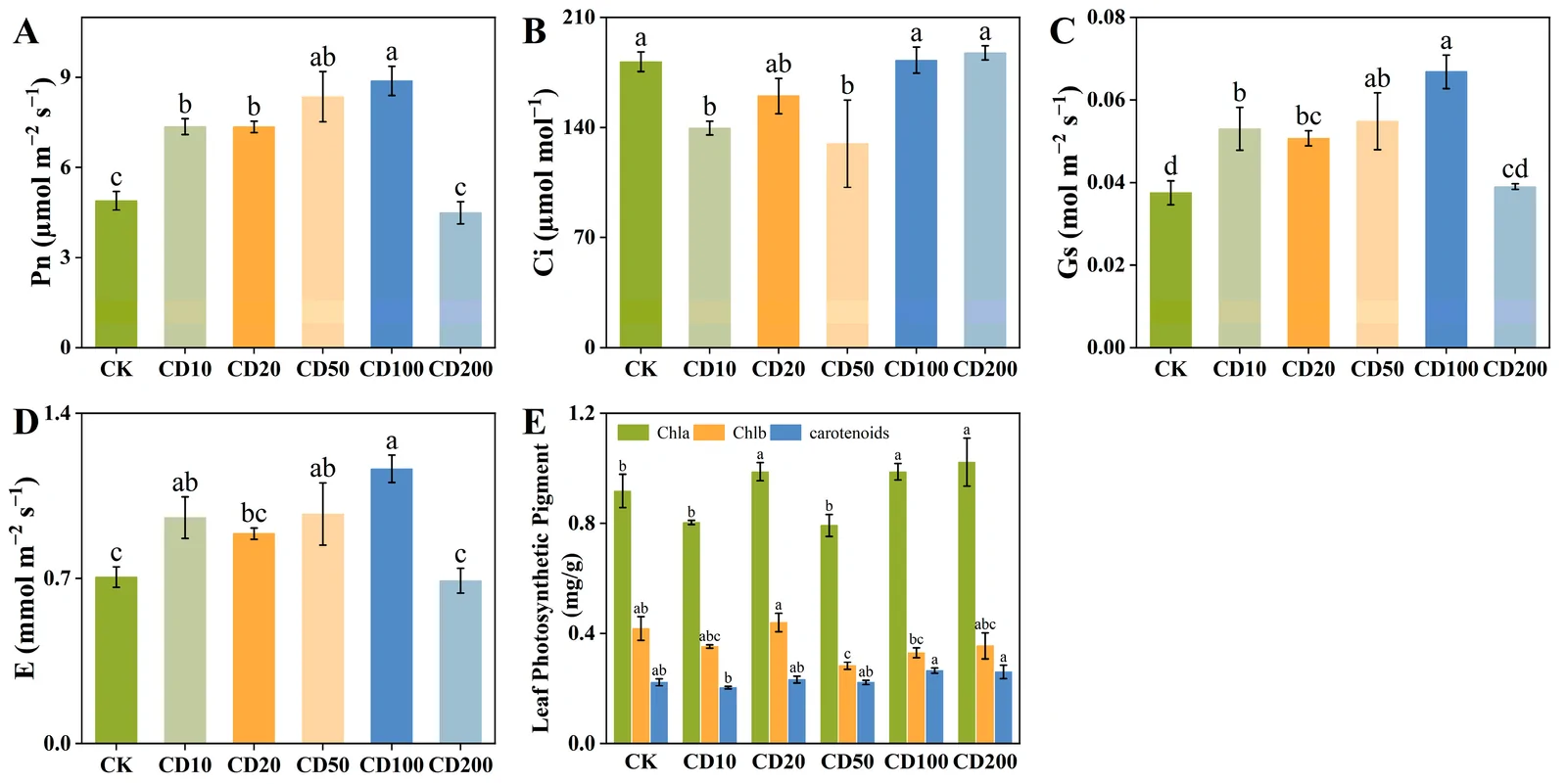
Effects of different concentrations of CDs on photosynthetic parameters and photosynthetic pigments in cotton. (**A**) The net photosynthetic rate, Pn; (**B**) intercellular CO_2_ concentration, Ci; (**C**) stomatal conductance, Gs; (**D**) transpiration rate, E; (**E**) photosynthetic pigments (Chla: Chlorophyll a; Chlb: Chlorophyll b; Carotenoids). Error bars represent mean ± standard error (*n* = 4), and lowercase letters indicate significant differences at the *p* < 0.05 level.

CDs at different concentrations affected photosynthetic parameters in cotton plants, including net photosynthetic rate (Pn), intercellular CO_2_ concentration (Ci), stomatal conductance (Gs), and transpiration rate (E). Foliar application of appropriate CDs concentrations to cotton plants was beneficial for increasing Gs, E, and Pn (Figure 3A–D). After foliar application of CDs at 10, 20, 50, and 100 mg·L^−1^, Pn, Gs, and E all increased. In particular, under 100 mg·L^−1^ treatment, these parameters increased significantly, with Pn, Gs, and E increasing by 81.72%, 78.13%, and 64.92%, respectively, while Ci showed no significant change. Similarly, CDs also affected the contents of photosynthetic pigments. Figure 3E shows the effects of CDs on chlorophyll and carotenoids. Compared with the control group, the photosynthetic pigments responded differently to CDs: chlorophyll a showed a somewhat complex pattern, with an increasing trend under 20, 100, and 200 mg·L^−1^; chlorophyll b content was comparable to the CK only under 20 mg·L^−1^ treatment, while it was lower than the control group at all other concentrations; carotenoid content differed among the treatments, with the highest numerical value observed under the CD100 treatment. Chlorophyll a concentration grew by 7.63% following the 100 mg·L^−1^ CDs treatment, accompanied by a 19.10% enhancement in carotenoids. CDs addition promoted the accumulation of photosynthetic pigments in cotton leaves, thereby enhancing photosynthesis. Based on the overall responses of the measured photosynthetic parameters and photosynthetic pigments relative to CK, CD100 was selected for further analysis under the conditions tested.

**Figure 4:**
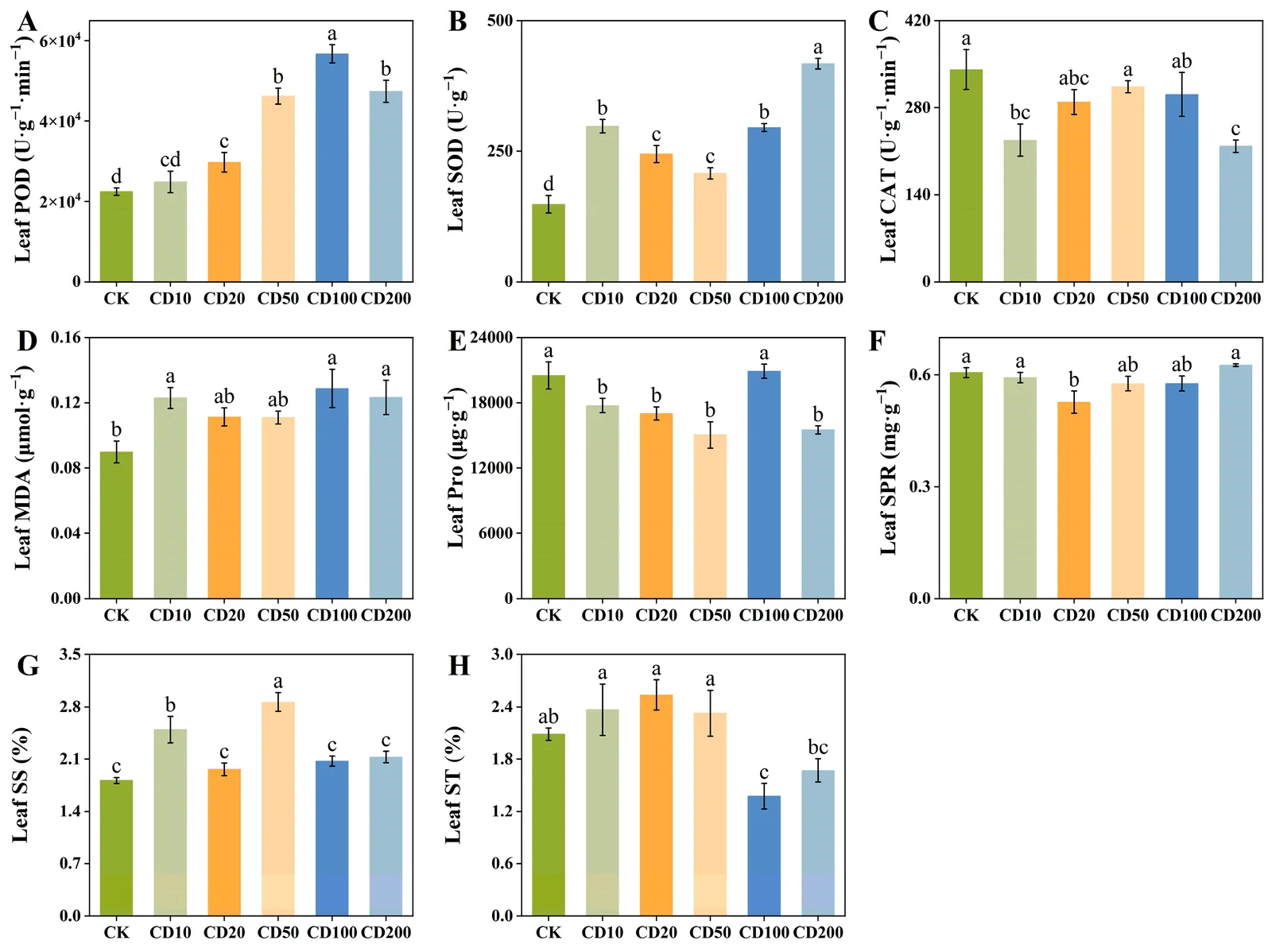
Effects of different concentrations of CDs on cotton physiological and biochemical indicators. (**A**) Leaf POD enzyme activity; (**B**) leaf SOD enzyme activity; (**C**) leaf CAT enzyme activity; (**D**) leaf MDA; (**E**) leaf proline; (**F**) leaf soluble protein; (**G**) leaf soluble sugar; (**H**) leaf soluble starch. Error bars represent mean ± standard error (*n* = 4), and lowercase letters indicate significant differences at the *p* < 0.05 level.

CDs modulated the antioxidant defense of cotton (Figure 4A–D). POD, SOD, and CAT are important antioxidant enzymes in plants and can serve as defense substances to improve plant antioxidant capacity. Compared with that of the control group, POD activity rose initially but declined later in response to rising CDs concentrations, peaking at 100 mg·L^−1^ with a 152.97% increase. All CDs concentrations significantly enhanced SOD activity relative to the CK, showing a 99.30% increase at 100 mg·L^−1^ CDs. Compared with the CK, CAT activity decreased in all treatments, with an 11.73% reduction under the 100 mg·L^−1^ CDs treatment. Leaf MDA content increased significantly with rising CDs concentration. Relative to the CK, MDA content increased in all treatment groups, and under the CD100 treatment, it increased by 43.43%. The results for organic osmotic regulators showed that soluble sugar, soluble starch, proline, and soluble protein exhibited different trends (Figure 4E–H). Compared with that of the control group, proline content did not change significantly under CD100 treatment, increasing by only about 1.94%. SPR content was significantly lower under CD20 than under CK, CD10, and CD200, whereas no other significant pairwise differences were detected. Soluble sugar content was highest under CD50, which differed significantly from the other treatments. Although the soluble sugar content under CD100 was 14.31% higher than that under CK, this difference was not statistically significant. Soluble starch content showed no consistent concentration-dependent decrease. It was significantly lower under CD100 than under CK, CD10, CD20, and CD50, while the difference between CD100 and CD200 was not significant; compared with that of the control group, ST decreased by 34.16% under CD100 treatment.

As shown in Figure S2, CDs also affected the physiological and biochemical responses of cotton roots. In terms of antioxidant defense, CD100 treatment significantly affected the antioxidant enzyme activities in roots (Figure S2A–C), elevating the activities of both POD (by 30.57%) and CAT (by 55.01%) relative to the control. However, root MDA content reached the maximum under the CD100 treatment, indicating that CDs may have caused pronounced lipid peroxidation and oxidative stress in roots (Figure S2D). Regarding osmotic adjustment substances, CD100 treatment, compared with CK, significantly boosted the levels of proline and soluble sugar (Figure S2E,G), resulting in 34.27% and 70.27% increments, respectively. In addition, root soluble protein content showed no significant change (Figure S2F), whereas soluble starch levels decreased significantly by 31.82% (Figure S2H) compared with CK. Although the physiological and biochemical parameters did not all exhibit strictly monotonic responses across the tested concentrations, the integrated results showed that CD100 produced the greatest increases in fresh and dry weights. With additional support from the physiological and biochemical data, CD100 was therefore considered the most appropriate concentration for promoting cotton growth under the present experimental conditions.

### 2.3. Metabolomic Profiling of Cotton

**Figure 5:**
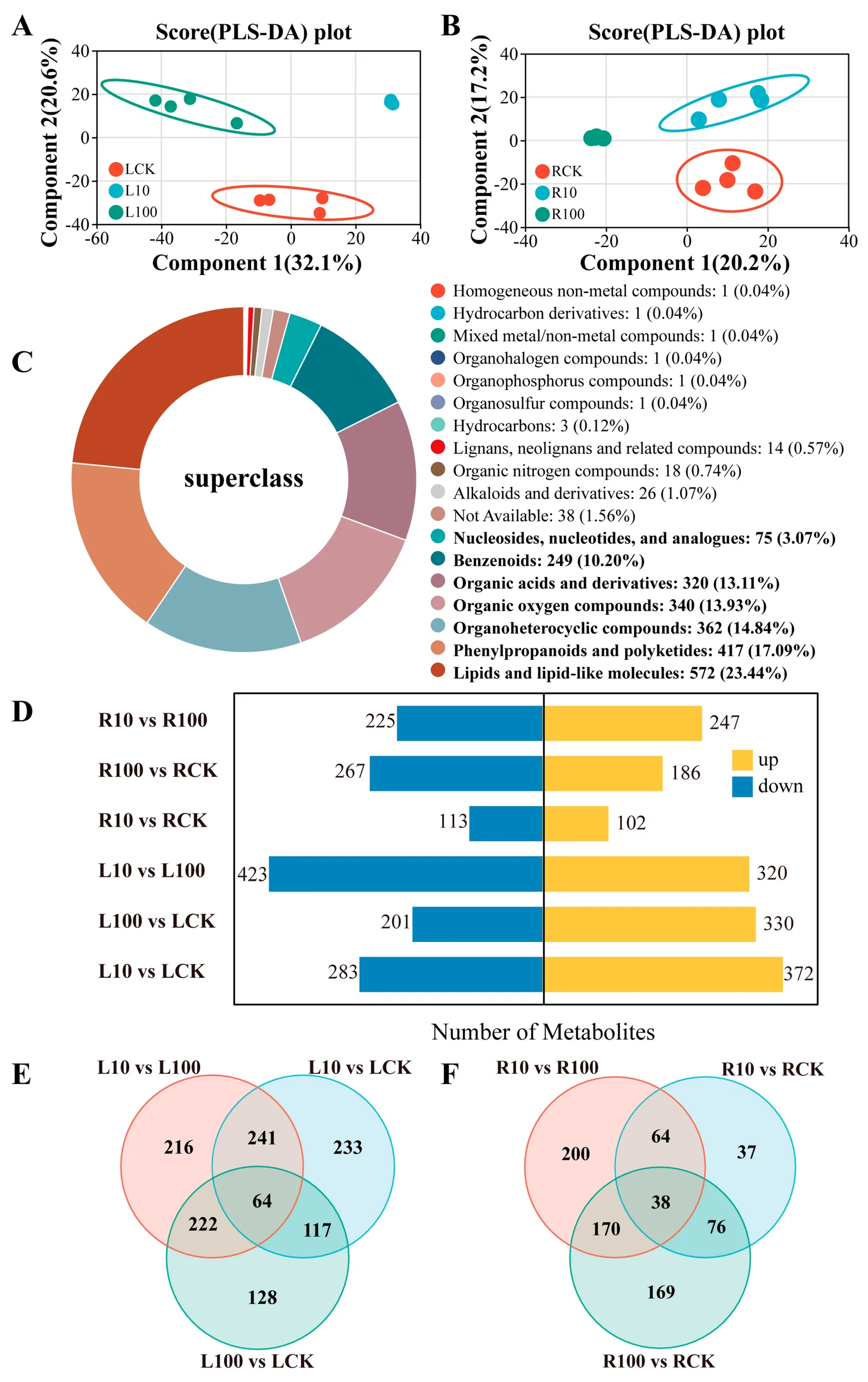
Metabolomic analysis of cotton. (**A**) PLS-DA of leaf samples; (**B**) PLS-DA of root samples; (**C**) classification characteristics of substances; (**D**) comparison of the number of DAMs between groups. Yellow represents upregulated DAMs, and blue indicates downregulated DAMs; (**E**) Venn diagram of DAMs between comparison groups in leaves; (**F**) Venn diagram of DAMs between comparison groups in roots.

To elucidate the effects of CDs on cotton metabolites, an untargeted metabolomics analysis was performed on cotton leaf and root tissues. To assess global metabolic profiles among the different treatments, principal component analysis (PCA) was applied. The leaf PCA (PC1: 31.90%, PC2: 17.80%; Figure S3A) demonstrated a clear separation between the CK and the CD-treated groups. Similarly, the root PCA (PC1: 33.90%, PC2: 14.00%; Figure S3B) segregated the CD100 group from the remaining treatments, confirming treatment-induced metabolic alterations. Further validation using the PLS-DA model demonstrated significant differences in metabolites between groups (Figure 5A,B). Additionally, the Pearson correlation coefficient was employed to assess the homogeneity within replicate groups. A correlation coefficient closer to 1 indicates greater consistency in metabolite composition and levels across samples, as well as better uniformity. The results showed that the correlation coefficients among leaf samples were all above 0.88, and those among root samples were all above 0.93 (Figure S3C,D), indicating good sample stability and high overall quality of the experimental data.

### 2.4. Identification of DAMs and Enrichment Analysis of Metabolic Pathways

Based on the HMDB database, a total of 2440 metabolites were annotated (Table S2). As shown in Figure 5C, these metabolites were grouped into 18 distinct categories. Among them, lipids and lipid-like molecules (572, 23.44%) represented the most abundant class, followed by phenylpropanoids and polyketides (417, 17.09%), organoheterocyclic compounds (362, 14.84%), organic oxygen compounds (340, 13.93%), and organic acids and derivatives (320, 13.11%). To further investigate metabolic differences among cotton groups, DAMs were analyzed, and their numbers in each comparison are shown in Figure 5D. In leaf tissues, L10 vs. LCK identified 655 DAMs (372 upregulated and 283 downregulated), L100 vs. LCK identified 531 DAMs (330 upregulated and 201 downregulated), and L10 vs. L100 identified 743 DAMs (320 upregulated and 423 downregulated). In root tissues, R10 vs. RCK identified 215 DAMs (102 upregulated and 113 downregulated), R100 vs. RCK identified 453 DAMs (186 upregulated and 267 downregulated), and R10 vs. R100 identified 472 DAMs (247 upregulated and 225 downregulated). Venn diagrams were utilized to delineate the specific and overlapping DAMs across different groups. In leaf tissues, 1221 DAMs were identified under the three treatments, with 64 shared by all of them (Figure 5E). Similarly, root tissues yielded 754 DAMs, including 38 common to all three treatments (Figure 5F).

**Figure 6:**
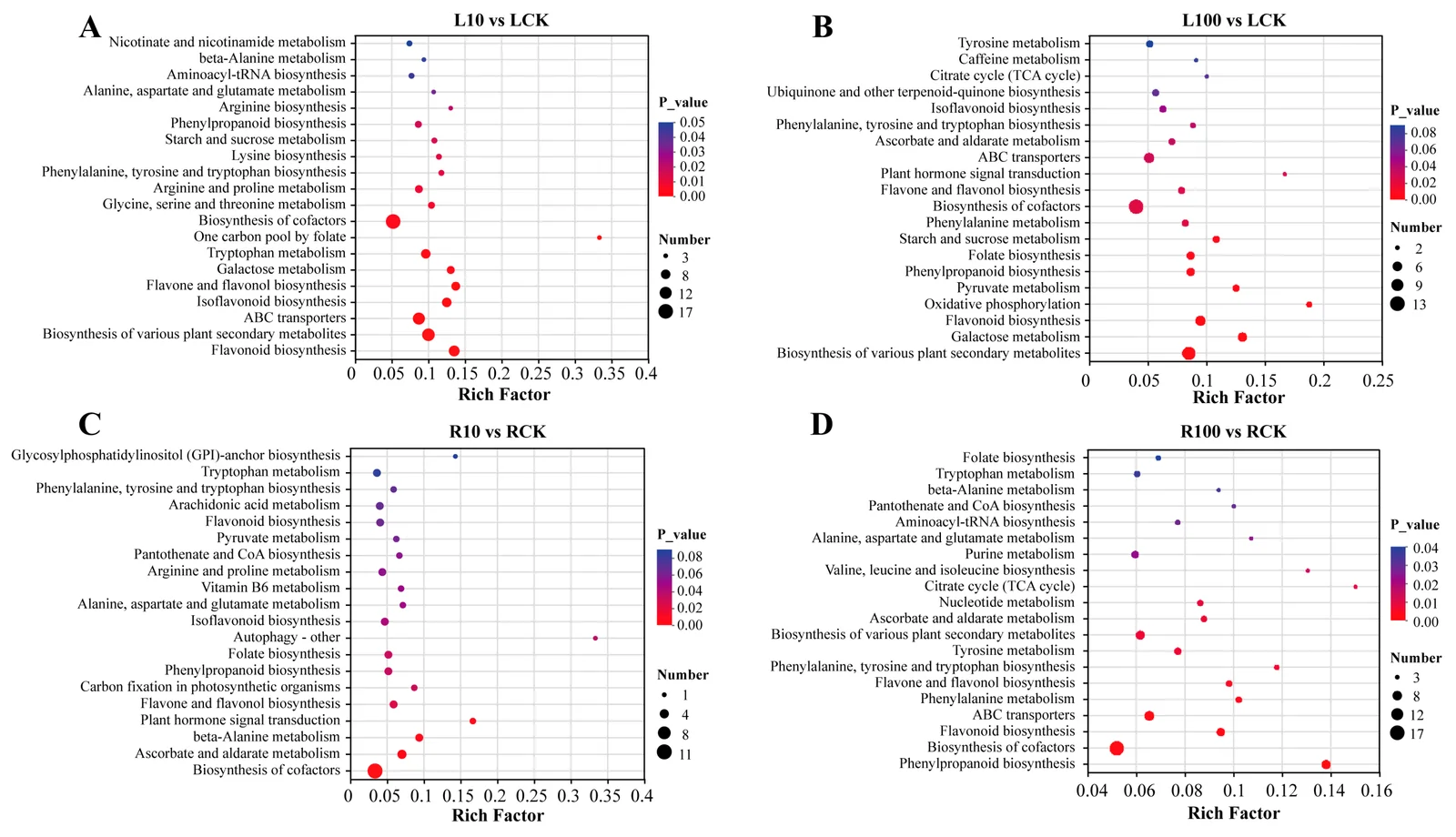
KEGG pathway enrichment of DAMs. (**A**) L10 vs. LCK; (**B**) L100 vs. LCK; (**C**) R10 vs. RCK; (**D**) R100 vs. RCK.

For a comparative analysis of metabolic profiles under CDs treatments at different concentrations, DAMs from both root and leaf tissues were subjected to KEGG pathway enrichment. The results revealed that these DAMs across different tissues and treatments were primarily involved in the biosynthesis of cofactors, flavonoid biosynthesis, flavone and flavonol biosynthesis, as well as phenylalanine, tyrosine and tryptophan biosynthesis. Within leaf samples, the differentially enriched pathways in the L10 vs. LCK comparison predominantly involved flavonoid biosynthesis, biosynthesis of various plant secondary metabolites, ABC transporters, isoflavonoid biosynthesis, and flavone and flavonol biosynthesis (Figure 6A; Table S3). Comparing L100 with LCK revealed marked pathway enrichment in biosynthesis of various plant secondary metabolites, galactose metabolism, flavonoid biosynthesis, and oxidative phosphorylation (Figure 6B; Table S4). In roots, pathways showing significant enrichment in the R10 vs. RCK comparison included biosynthesis of cofactors, ascorbate and aldarate metabolism, beta-alanine metabolism, and plant hormone signal transduction (Figure 6C; Table S5). For R100 vs. RCK, however, the enriched pathways shifted towards phenylpropanoid biosynthesis, biosynthesis of cofactors, flavonoid biosynthesis, and ABC transporters (Figure 6D; Table S6). Overall, CDs exposure induced significant alterations in secondary metabolism, signal transduction, as well as transmembrane transport across leaf and root tissues, with higher concentrations triggering broader metabolic reprogramming in cotton.

### 2.5. Correlation Analysis Between DAMs and Growth Parameters

**Figure 7:**
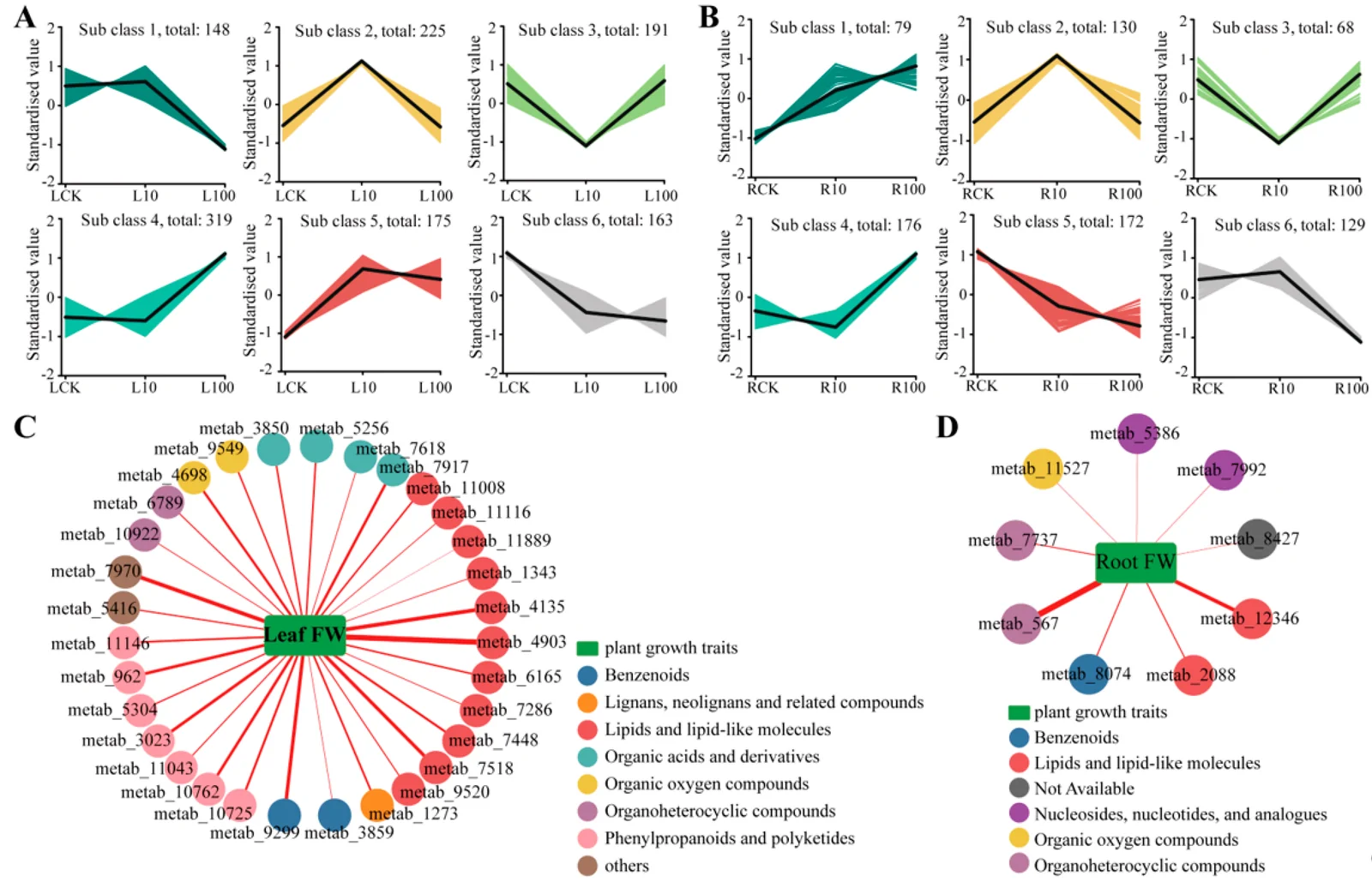
Characteristic metabolite analysis. (**A**) K-means clustering of leaf DAMs (k = 6); (**B**) K-means clustering of root DAMs (k = 6); (**C**) correlation network between leaf DAMs and leaf fresh weight; (**D**) correlation network between root DAMs and root fresh weight. The red lines indicate a positive correlation, and the different circles represent different types of metabolites.

For the identification of treatment-specific metabolites, K-means clustering was conducted using the DAMs profiles of root and leaf samples. The 1221 DAMs in leaves and 754 DAMs in roots were each classified into six subclusters. The number of metabolites in each subcluster was as follows: in leaves, the six subclusters contained 148, 225, 191, 319, 175, and 163 metabolites, respectively (Figure 7A; Table S7). In roots, the corresponding numbers were 79, 130, 68, 176, 172, and 129 (Figure 7B; Table S8). In leaf tissue, subcluster 4 contained 319 metabolites. Metabolites in this cluster showed low abundance under the LCK and L10 treatments, but were sharply and strongly upregulated under the high-dose L100 treatment. This dose-dependent, monotonically increasing pattern indicates that the metabolites in subcluster 4 are specific biomarkers of the L100 treatment and that their dynamic changes are of important biological significance. Therefore, this cluster represents the most important candidate metabolite set for investigating the mechanism underlying the high-dose effects of L100, and the metabolites within this cluster should be prioritized for further analysis. Correspondingly, in root tissue, the metabolites in subclusters 1 and 4 exhibited similar trends, and were therefore considered key metabolites that may play important roles under 100 mg·L^−1^ CDs treatment.

According to the evaluated growth parameters, CDs showed a clear promoting effect on plant growth (Figure 2). To explore the interplay between DAMs and plant growth, we further performed a correlation analysis between the key metabolites obtained from K-means clustering and the growth parameters of cotton, thereby providing an intuitive visualization of their associations. The network diagram (Figure 7C,D) showed that leaf FW was significantly correlated with multiple metabolites, which primarily fell under lipids and lipid-like molecules, followed by phenylpropanoids and polyketides. Of these, metab_4903 (Gibberellin A7, GA7) and metab_4135 (Methyl dihydrojasmonate, MDJ) showed significant positive correlations with leaf FW, with both belonging to the former category. Root FW was significantly associated with metabolites classified as “organoheterocyclic compounds”, alongside “lipids and lipid-like molecules”. In particular, metab_567 (2(5H)-Furanone), an organoheterocyclic compound, correlated positively and significantly with root FW. These findings indicate that these metabolic changes are closely associated with plant growth, and that the metabolites differ among various tissues.

## 3. Discussion

### 3.1. Dose-Dependent Promotion of Cotton Growth by CDs

The results demonstrated that the regulation of cotton growth by CDs exhibited a typical dose-dependent pattern, showing a promotive effect at certain doses, with the optimal promotion observed at 100 mg·L^−1^, followed by a weakened effect at higher concentrations, such as 200 mg·L^−1^. This pattern is partially consistent with the commonly observed “low-dose stimulation and high-dose inhibition” response [21,22]. However, it may also indicate that CD200 exceeded the optimal effective concentration range for CDs-mediated growth promotion, resulting in the loss of the significant promotive effect observed at CD100. Under the CD100 treatment, the FW and DW of the leaves and roots increased significantly compared with those of the control, indicating that CDs promoted biomass accumulation in these organs. The specific leaf area increased significantly by 52.51%. Although SLA is positively associated with plant growth rate in some comparative studies [23,24], the increase in SLA observed under the CDs treatment should be interpreted primarily as a leaf-level morphological response rather than as direct evidence of enhanced whole-plant growth. Meanwhile, the increase in SLA endowed cotton leaves with a larger photosynthetic area, enabling greater capture of light energy and thereby enhancing photosynthetic efficiency. However, plant height did not differ significantly among the treatment groups, which contrasts with the broad growth-promoting effects commonly reported for some nanomaterials [25]. As a macroscopic indicator of cell elongation and meristematic activity, the stability of plant height may suggest that the primary effects of CDs were more closely associated with leaf morphological adjustment and organ-specific biomass accumulation, particularly in the leaves and roots, rather than with stem elongation or overall longitudinal growth [22]. Therefore, although more biomass accumulated in the plants, this biomass may not have been preferentially allocated to stem elongation. In summary, these findings indicate that CDs at the optimal concentration can effectively convert environmental resources into biomass, highlighting their great potential as a novel plant growth regulator.

### 3.2. CDs Enhance Photosynthetic Efficiency and Pigment Regulation in Cotton

Photosynthesis critically governs biomass accumulation and is vital to plant growth and agriculture [26]. Crop growth is closely associated with photosynthetic efficiency. In alignment with this, earlier investigations indicate that CDs can improve crop photosynthesis and further promote growth [27,28]. Application of 100 mg·L^−1^ CDs to cotton markedly increased Pn, Gs, and E, confirming that CDs-induced growth enhancement is driven by elevated photosynthetic efficiency [29]. Elevations in Pn are typically ascribed to either stomatal or non-stomatal factors. The significant increase in Gs indicates greater stomatal opening, which facilitates CO_2_ entry into the leaves. However, there was no significant change in Ci, further suggesting that the increase in Pn was driven not only by stomatal opening but also by the alleviation of non-stomatal limitations, namely enhanced CO_2_ utilization efficiency in mesophyll cells [30]. Nanomaterials have been documented to stimulate photosynthesis by increasing electron transfer efficiency or optimizing photosystem II (PSII) activity, which may explain the capacity of CDs to elevate CO_2_ utilization [31]. In this study, photosynthetic enhancement was directly associated with improved stomatal conductance. Nonetheless, whether CDs’ optical and electronic properties complementarily promote light harvesting and electron transfer requires further investigation.

For photosynthetic pigments, a substantial elevation in chlorophyll a and carotenoid levels was elicited by 100 mg·L^−1^ CDs. Chlorophyll directly correlates with photosynthesis and acts as a major estimator of photosynthetic capacity, which critically governs plant growth. The increase in chlorophyll a directly enhances light-harvesting capacity. The significant accumulation of carotenoids has dual implications: on the one hand, they function as supplementary light-absorbing molecules and optimize photon utilization; on the other hand, they provide photoprotective effects and act as critical components of non-photochemical quenching (NPQ) under high-light or stress environments, which shields chlorophyll from oxidative impairment [32,33]. The changes in chlorophyll and carotenoid contents may be associated with the photoluminescent properties of CDs, which have been reported to influence light harvesting and photosynthetic performance. However, because the photophysical effects of CDs were not directly examined in this study, this potential association remains speculative and requires further validation. Such differential pigment responses may reflect a fine-tuning of the photosynthetic antenna in cotton, aiming to promote photosynthesis while simultaneously enhancing photoprotection to cope with mild environmental changes or oxidative stress potentially induced by CDs [34].

### 3.3. CDs Regulate Oxidative Stress and Osmotic Adaptation in Cotton

As described above, this study observed changes in antioxidant enzyme activities and the MDA content, revealing that CDs activated the antioxidant defense system of cotton during growth promotion. Under 100 mg·L^−1^ CDs treatment, leaf POD and SOD activities were significantly enhanced, whereas CAT activity decreased. Within this defense network, the primary elimination of superoxide anion radicals (·O_2_^−^) is mediated by SOD, which yields H_2_O_2_ for subsequent scavenging by POD. The simultaneous and substantial increase in these two enzyme activities suggests that CDs induced a slight elevation in intracellular reactive oxygen species (ROS) levels, which rapidly triggered an efficient and proactive ROS scavenging mechanism in cotton [35,36]. CAT is another crucial enzyme involved in H_2_O_2_ clearance. However, during certain environmental stresses, cells tend to scavenge H_2_O_2_ through POD and the ascorbate-glutathione cycle while suppressing CAT activity. This process retains a certain amount of H_2_O_2_ to function as a signaling molecule, triggering defense gene expression and modulating growth [37]. Factors such as plant species, nanomaterial characteristics, treatment period, and applied dose critically determine the variations in antioxidant enzyme activities within plants exposed to nanoparticles [38]. Since MDA is a primary outcome of lipid peroxidation, its accumulation is widely accepted as an indicator of membrane impairment or oxidative stress [39]. Our data revealed a marked elevation in MDA levels across all CDs-treated groups. The concurrent elevation of MDA content and POD/SOD activities indicates that CDs indeed induced oxidative stress in cells, but this stress was mild and manageable [40,41]. Such an outcome is likely due to the acceleration of ROS synthesis triggered by CDs during cellular metabolic processes. Although elevated ROS levels led to higher MDA accumulation, they also activated a strong antioxidant defense response, enabling cotton to cope with the stress and convert ROS into signaling molecules, ultimately promoting growth [42,43]. The response observed at the highest CDs concentration (CD200) suggests that the beneficial effects of CDs may be subject to a concentration-dependent threshold. At this concentration, SOD activity reached its maximum, whereas CAT activity decreased, indicating a possible imbalance in the coordinated detoxification of ROS. The further accumulation of MDA may reflect aggravated oxidative damage and impaired membrane stability. These findings suggest that excessive CDs application may shift the plant response from a mild and manageable oxidative signal to a more severe stress condition. At appropriate doses, CDs may induce mild oxidative stress in plants, accompanied by increased MDA levels and further activation of enzymatic antioxidant defenses. By enhancing their antioxidant capacity to alleviate oxidative damage and adapt to external stress, plants may exhibit a “stress-induced growth” phenomenon under certain conditions, which is a common mode of action of nanomaterials in plants [44].

Osmolytes in plant cells increase intracellular solute concentrations to maintain osmotic balance, thereby enabling plants to adapt to various environmental stresses. In plant systems, organic osmoregulators are among the major osmotic solutes, which chiefly consist of soluble carbohydrates, proline, and soluble proteins [45]. Compared with the CK group, proline and soluble protein contents did not change significantly under 100 mg·L^−1^ CDs treatment. Although the elevated MDA levels indicated that the plants experienced a certain degree of oxidative stress, this stress was not sufficient to significantly induce the synthesis of osmoprotectants, including proline, suggesting that this response induced by CDs may have been mainly confined to ROS-related signaling, without further developing into a pronounced osmotic adjustment response [46]. Soluble starch is an important carbohydrate in cotton and serves as a link between photosynthesis and biomass accumulation [47,48]. Such a substantial reduction in starch reserves indicates that it can be rapidly degraded and converted into soluble sugars, which were quickly transported and utilized to support biomass accumulation and metabolic activity [49]. Soluble sugars are not only important osmotic regulators and signaling molecules in plants, but also essential energy and carbon sources for other physiological processes [50]. Their accumulation may be associated with the increased Pn and accelerated starch hydrolysis, indicating enhanced carbon assimilation capacity in plants, thereby providing sufficient energy and material resources for growth and development [51,52].

### 3.4. CDs Reprogram the Metabolome of Cotton Roots and Leaves

Metabolomic analysis revealed that CDs treatment induced hundreds of DAMs in leaves and roots, indicating that the regulatory effects of CDs involve extensive reprogramming of metabolic pathways. Lipids and lipid-like molecules, phenylpropanoids and polyketides, along with organoheterocyclic compounds, represented the majority of the metabolites identified. The high proportion of lipids and lipid-like molecules, which are key cell membrane components mediating lipid signaling and energy storage, may reflect changes in cell membrane structure and associated signaling networks [53]. Meanwhile, phenylpropanoids and polyketides, as important components of plant secondary metabolism, are closely associated with antioxidant activity, defense, and adaptive responses, suggesting that plants may have enhanced relevant defensive metabolic pathways to cope with adverse external conditions [54].

KEGG enrichment analysis further supported the mechanism of CDs at the pathway level. Compared with low concentration treatment, high concentration treatment induced more extensive metabolic reprogramming, and this effect was clearly observed in both leaves and roots. Although the enriched pathways differed somewhat between tissues, phenylpropanoid biosynthesis and flavonoid biosynthesis served as key pathways notably overrepresented in both L100 vs. LCK and R100 vs. RCK comparisons. These results suggest that CDs may activate the plant secondary metabolic network in response to external stimuli, thereby inducing metabolic reprogramming associated with defense and stress adaptation. The phenylpropanoid biosynthesis pathway is a central hub for generating lignin and flavonoids [55], which serve as important antioxidants and defensive compounds in plants. They not only possess strong antioxidant activity but also participate in processes such as scavenging ROS, preventing lipid peroxidation, and resisting pathogens [56,57]. This enrichment profile aligns well with the augmented POD/SOD activities and MDA accumulation, indicating that cotton may respond to CDs-induced external stimulation by enhancing defensive secondary metabolism, promoting redox homeostasis, and strengthening chemical and structural defense barriers. In addition, the enrichment of the oxidative phosphorylation pathway in leaves suggests that CDs treatment may enhance mitochondrial energy metabolism, thereby providing the ATP required for defense responses, metabolic reprogramming, and cellular homeostasis maintenance [58]. This change is consistent with the elevated Pn, augmented enzyme activities, and accelerated growth phenotype observed in the results, suggesting that leaves may meet the metabolic demands induced by CDs through an enhanced energy supply capacity. The enrichment of the ABC transporters pathway in roots further suggests that cotton may regulate the transport and accumulation of secondary metabolites (including flavonoids, phenolic acids, and other phenylpropanoid derivatives) by enhancing transmembrane transport capacity, thereby maintaining intracellular homeostasis and supporting adaptive root remodeling [59,60]. Taken together, CDs-triggered metabolic alterations are not confined to a single defense pathway but instead represent a coordinated adaptive response network in cotton through the synergistic interaction of secondary metabolite biosynthesis, energy metabolism, and ATP-dependent transport systems.

### 3.5. Relationship Between Metabolites and Plant Growth

Among the key metabolites significantly correlated with FW of cotton leaves and roots, several compounds with potential physiological regulatory functions were identified, including gibberellin A7, methyl dihydrojasmonate (MDJ), and 2(5H)-furanone. Gibberellins are important natural plant hormones that modulate nearly all aspects of the plant life cycle [61,62]. GA7 is a bioactive gibberellin that promotes plant development primarily by driving cell elongation and organ formation, although its effects vary significantly among different species and tissues [63,64]. Putrescine promotes tomato growth by elevating the endogenous levels of auxin (IAA) and gibberellins (GA4 and GA7) [65]. Consistent with this finding, GA7 was positively correlated with cotton fresh weight in this study, suggesting that CDs treatment may promote leaf growth and biomass accumulation by enhancing gibberellin-related signaling pathways. Methyl dihydrojasmonate is not a conventional endogenous growth hormone, but as an exogenous metabolite related to jasmonic acid, it can significantly influence plant physiological metabolism and defense responses. MDJ has been reported to promote saponin biosynthesis in *Panax notoginseng* and upregulate the expression of multiple genes involved in saponin biosynthesis [66]. Comprehensive analyses of *Paris polyphylla* var. *yunnanensis* treated with MDJ further showed that MDJ not only increased photosynthetic pigment content, antioxidant enzyme activity, and osmolyte levels, but also enhanced stress tolerance [67]. Overall, MDJ is more appropriately regarded as a jasmonic acid-like regulatory molecule, whose primary functions include promoting secondary metabolism, enhancing stress resistance, and alleviating chemical stress rather than directly acting as a biomass-promoting factor. Our analysis also revealed that 2(5H)-Furanone was significantly positively correlated with cotton root FW, although direct evidence for its physiological function in plants remains limited. Previous studies have shown that derivatives of the 2(5H)-Furanone scaffold do not exclusively exhibit growth-inhibitory effects; instead, their bioactivities are highly dependent on both species and structure. For example, nostoclide analogues exhibited differential effects in different plant systems: they inhibited root growth in *Lolium multiflorum* but promoted root growth in *Physalis ixocarpa* [68]. This suggests that such compounds possess diverse biological effects in plants, with clear structure- and species-dependent characteristics. In summary, the cotton growth enhancement elicited by CDs is mediated by metabolic shifts in numerous key compounds. These metabolites show distinct responses across different tissues, suggesting that CDs-mediated metabolic reprogramming exhibits significant tissue specificity and may synergistically regulate cotton growth through multiple metabolic pathways.

## 4. Materials and Methods

### 4.1. Synthesis and Characterization of CDs

The preparation of CDs adhered to the literature-reported strategy [69], and the detailed experimental procedure is available in the Supplementary Materials. Subsequently, the obtained CDs underwent comprehensive physicochemical profiling. Specifically, a transmission electron microscope (TEM, JEOL JEM-2100F, JEOL, Tokyo, Japan) was harnessed to probe the structure and dimensions, while the elemental constituents and surface chemistry were evaluated using an X-ray photoelectron spectrometer (XPS, Thermo Fisher Scientific, USA) and a Fourier transform infrared spectrometer (FTIR, VERTEX 70 RAMI, Bruker, Ettlingen, Germany). Detailed information on the specifications, model numbers, and manufacturers of the instruments used is provided in Table S1.

### 4.2. Cotton Cultivation and Experimental Conditions

The cotton cultivar “Xinluzao 57” served as the research subject, with plants grown in pots under soil culture conditions (20 cm diameter pots, 2.5 kg soil per pot). Prior to sowing, the ginned seeds were disinfected with 10% H_2_O_2_ for 1 h, rinsed with sterile water and soaked for 12 h. Seed germination was conducted under dark conditions at 28 °C (12–24 h). Following successful germination and transplantation, the seedlings were grown for 15–30 days under ambient outdoor climatic conditions to ensure robust growth and acclimatization. The plants were manually irrigated every two days, with additional irrigation provided when necessary to prevent water stress. They were then randomly assigned to different experimental groups, with two cotton plants grown in each pot. CDs treatment was initiated at the seedling stage, before bud formation, and the plants were maintained outdoors under the same natural conditions until the end of the experiment.

The pot experiment included six treatment groups: 0 (CK), 10 (CD10), 20 (CD20), 50 (CD50), 100 (CD100), and 200 (CD200) mg·L^−1^, respectively, with four biological replicates per group. To facilitate effective absorption of the CDs, a foliar spray application method was employed [70]. The CD suspension was prepared in deionized water and thoroughly dispersed by ultrasonic treatment for 15 min prior to use. A total volume of 5 mL per pot was applied using a handheld micro-sprayer from a distance of approximately 15–20 cm. The spray was directed onto both the adaxial and abaxial leaf surfaces until complete and uniform foliar wetting was achieved, while avoiding excessive runoff into the soil. Control seedlings received 5 mL of deionized water in the same manner. For all groups, the foliar application was performed once daily for 10 consecutive days. When the plants reached 60 days after sowing, tissues from different plant parts were harvested, pretreated, and properly stored for subsequent physiological and biochemical measurements.

### 4.3. Assessment of Cotton Growth and Physiological Traits

Measurements of growth parameters: The fresh weights (FW) of cotton were determined with an analytical balance. Once inactivated and oven-dried, the samples were weighed to obtain their respective dry weights (DW). Plant height was measured with a measuring tape from the stem base to the apex. Meanwhile, foliar dimensions were determined by image analysis, with the specific leaf area (SLA) subsequently derived as the ratio of total leaf area to the corresponding dry biomass [24,71].

Measurement of photosynthetic parameters: Gas exchange parameters, specifically stomatal conductance (Gs), transpiration rate (E), intercellular CO_2_ concentration (Ci), and net photosynthetic rate (Pn), were quantified using the LI-6800 portable gas exchange system (LI-COR, Lincoln, NE, USA). Additionally, photosynthetic pigments (chlorophyll and carotenoids) were isolated via ethanol and subsequently evaluated through spectrophotometry [72].

Measurements of antioxidant system indicators: the activities of superoxide dismutase (SOD), peroxidase (POD), and catalase (CAT), together with malondialdehyde (MDA) content, were evaluated following established experimental guidelines [72].

Measurements of organic osmotic regulators: the acidic ninhydrin method for proline (Pro) [73]; the Bradford assay for soluble protein (SPR) [74]; and the anthrone colorimetric method for both soluble sugar (SS) and soluble starch (ST) [75].

### 4.4. Metabolomic Analysis

Metabolomic analysis was performed using 12 leaf samples and 12 root samples (derived from three treatment groups with four biological replicates each). Metabolomic profiling was performed using an LC-MS/MS platform (UHPLC-Exploris240, Thermo Fisher Scientific, Waltham, MA, USA); further technical specifications are available in the Supplementary Materials. The identification of differentially accumulated metabolites (DAMs) was constrained by a dual-threshold approach: variable importance in projection (VIP) values > 1 from the orthogonal partial least squares discriminant analysis (OPLS-DA) model and *p*-values < 0.05 obtained via Student’s *t*-test [76]. Subsequently, these DAMs were mapped to the KEGG database to elucidate the primary metabolic pathways involved. KEGG pathway enrichment analysis was performed using the hypergeometric test. For each pathway, the enrichment *p*-value was calculated based on the hypergeometric distribution, and multiple testing was corrected using the Benjamini–Hochberg method. Pathways with an adjusted *p*-value (FDR) < 0.05 were considered significantly enriched.

### 4.5. Statistical Analysis

Plant physiological and biochemical parameters are presented as mean ± SE (*n* = 4). Significant differences between groups were determined by one-way ANOVA and Duncan’s post hoc comparisons at *p* < 0.05. Graphs were produced with Origin 2024 and the ggplot2 package in the R environment (v4.3.2). Cytoscape (v3.10.3) served for network visualization. Furthermore, rank correlations were considered significant at |*r*| > 0.8 and *p* < 0.01.

## 5. Conclusions

This work provides a systematic insight into the dose-dependent effects of CDs on cotton growth and the underlying metabolic mechanisms by integrating morphological characteristics, physiological and biochemical indicators, and untargeted metabolomics. The results showed that CDs at different concentrations affected cotton growth, with 100 mg·L^−1^ identified as the most effective dose for growth promotion. At this concentration, CDs significantly enhanced shoot and root biomass, improved key photosynthetic parameters, and increased the physiological adaptability of cotton by activating antioxidant enzyme systems and modulating the levels of osmotic regulators. Metabolomic analysis further revealed that CDs treatment significantly altered the metabolic composition of cotton leaves and roots, and that higher treatment concentrations induced broader metabolic reprogramming. These changes were mainly enriched in pathways governing plant stress tolerance and growth, notably flavonoid biosynthesis and phenylpropanoid biosynthesis. Based on K-means clustering and correlation analysis, the identified characteristic metabolites may serve as potential biomarkers of the response to CDs and are closely associated with cotton biomass accumulation. In summary, CDs promote cotton growth by coordinately regulating physiological processes and key metabolic pathways, providing important physiological and metabolomic evidence for their agricultural application.

## Data Availability

The raw metabolomics data generated in this study are publicly available in the MetaboLights repository under accession number MTBLS15337.

## Supplementary Materials

The following supporting information can be downloaded at: https://www.mdpi.com/article/10.3390/plants15172659/s1, Figure S1. Particle size distribution histogram of CDs. Figure S2. Effects of different concentrations of CDs on physiological and biochemical indicators of cotton roots. (A) Root POD enzyme activity; (B) Root SOD enzyme activity; (C) Root CAT enzyme activity; (D) Root MDA; (E) Root proline; (F) Root soluble protein; (G) Root soluble sugar; (H) Root soluble starch. Error bars represent mean ± standard error (n = 4), and lowercase letters indicate significant differences at the *p* < 0.05 level. Figure S3. PCA analysis and Pearson correlation heatmap of metabolomic samples. (A) PCA of leaf samples (Ellipses show 95% confidence intervals for each group); (B) PCA of root samples (Ellipses show 95% confidence intervals for each group); (C) Pearson correlation analysis of leaf samples; (D) Pearson correlation analysis of root samples. Table S1. Characterization instruments used for CDs in this study. Table S2. Classification of HMDB Compounds. Table S3. KEGG pathways enriched for L10 vs. LCK. Table S4. KEGG pathways enriched for L100 vs. LCK. Table S5. KEGG pathways enriched for R10 vs. RCK. Table S6. KEGG pathways enriched for R100 vs. RCK. Table S7. K-means clustering analysis of differential metabolites in cotton leaves. Table S8. K-means clustering analysis of differential metabolites in cotton roots.

## Abbreviations

The following abbreviations are used in this manuscript:

CDs	Carbon dots
TEM	Transmission electron microscope
FTIR	Fourier transform infrared spectrometer
XPS	X-ray photoelectron spectrometer
FW	Fresh weight
DW	Dry weight
SLA	Specific leaf area
Chla	Chlorophyll a
Chlb	Chlorophyll b
Gs	Stomatal conductance
Ci	Intercellular CO_2_ concentration
Pn	Net photosynthetic rate
E	Transpiration rate
PSII	Photosystem II
NPQ	Non-photochemical quenching
POD	Peroxidase
SOD	Superoxide dismutase
CAT	Catalase
MDA	Malondialdehyde
ROS	Reactive oxygen species
Pro	Proline
SPR	Soluble protein
SS	Soluble sugar
ST	Soluble starch
DAMs	Differentially accumulated metabolites
GA7	Gibberellin A7
MDJ	Methyl dihydrojasmonate
VIP	Variable importance in projection
PCA	Principal component analysis
OPLS-DA	Orthogonal partial least squares discriminant analysis
