# Supplementary material for: Dose-Dependent Effects and Metabolomic Analysis of Foliar-Applied Carbon Dots on Cotton (Gossypium hirsutum L.) Growth
Source: Plants (Basel). 2026 Aug 30;15(17):2659. doi: 10.3390/plants15172659 (PMC13567299; doi:10.3390/plants15172659)
Supplement: Supplementary file 1 [file plants-15-02659-s001.zip › Supplementary Material-S2.pdf]

Supporting Information

Dose-dependent effects and metabolomic analysis of foliar-applied carbon dots on cotton growth

Qiong Wu[1,2], Wen Cao[1,2], Yudong Chen[1,2], Yuehan Liu[1,2], Eryang Li[1.2], Guanghui Lv[1,2,*]

1 College of Ecology and Environment, Xinjiang University, Urumqi 830046, China

2 Key Laboratory of Oasis Ecology of Education Ministry, Xinjiang University, Urumqi 830046, China

*Corresponding author: Guanghui Lv

E-mail address: guanghui_xju@sina.com

Address: College of Ecology and Environment, Xinjiang University, Urumqi 830046, China

Experimental Section

Preparation of CDs: CDs with good dispersibility and hydrophilicity were prepared from citric acid via a one-step microwave-assisted method. Specifically, 2.1 g of citric acid was dissolved in 20 mL of deionized water and dispersed uniformly. The mixture was then heated in a domestic microwave (P70D20TL-D4, Galanz, China) oven at 350 W until a yellow colloidal substance formed. After cooling to room temperature, the colloidal product was redispersed in deionized water, filtered through a 0.22 μm membrane, and then lyophilized under vacuum to obtain the CDs sample.

Detailed protocol for metabolomic analysis: The extraction, detection, and quantification of metabolites were conducted by Shanghai Majorbio Bio-pharm Biotechnology Co., Ltd. (Shanghai, China). The LC-MS/MS analysis of sample was conducted on a UHPLC-Orbitrap Exploris 240 system equipped with an ACQUITY HSS T3 column (100 mm × 2.1 mm i.d., 1.8 μm; Waters, USA) The LC-MS raw data were preprocessed using Progenesis QI software (Waters Corporation, Milford, USA), and a data matrix containing retention time, mass-to-charge ratio, and peak intensity was obtained for subsequent analysis. The MS and MS/MS spectra were matched against HMDB entries for metabolite identification, and the identified metabolites were subsequently annotated using the KEGG database for pathway analysis.

Supplementary Figures

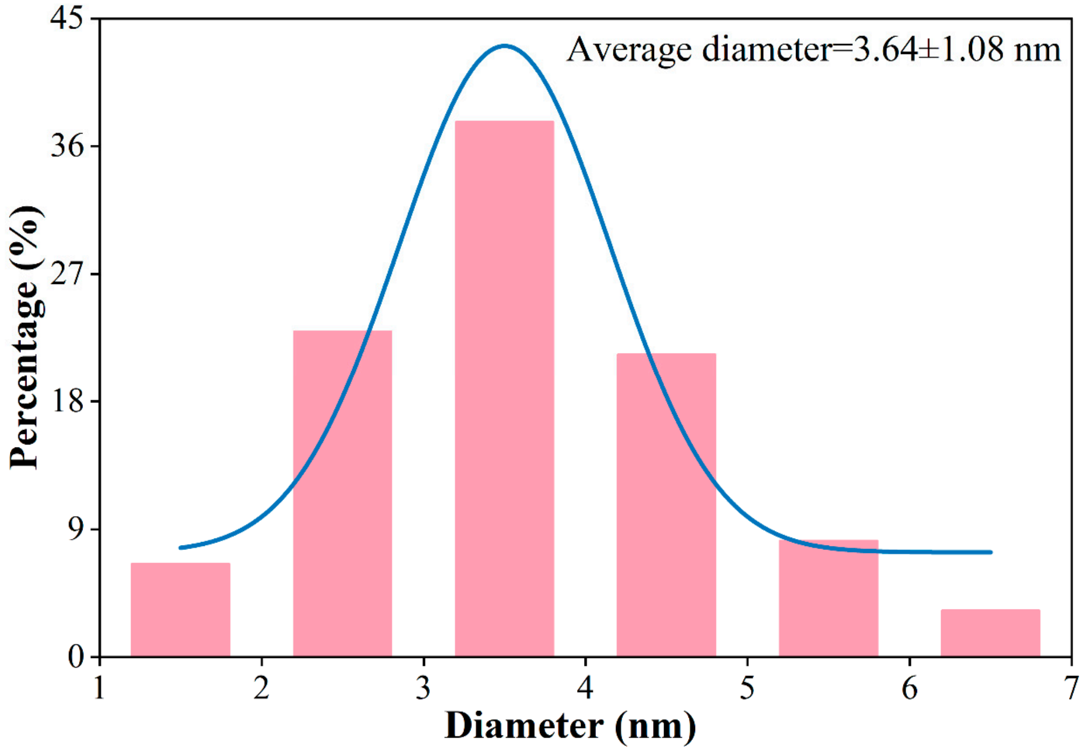

Figure S1. Particle size distribution histogram of CDs.

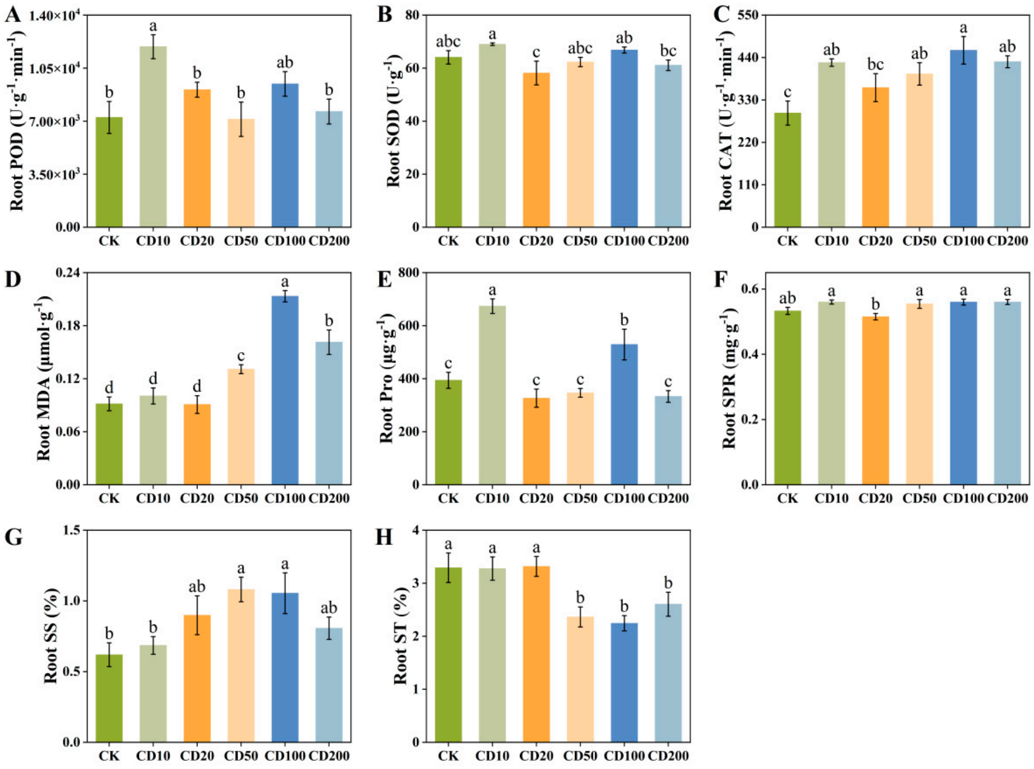

Figure S2. Effects of different concentrations of CDs on physiological and biochemical indicators of cotton roots. (A) Root POD enzyme activity; (B) Root SOD enzyme activity; (C) Root CAT enzyme activity; (D) Root MDA; (E) Root proline; (F) Root soluble protein; (G) Root soluble sugar; (H) Root soluble starch. Error bars represent mean ± standard error (n=4), and lowercase letters indicate significant differences at the $p<0.05$ level.

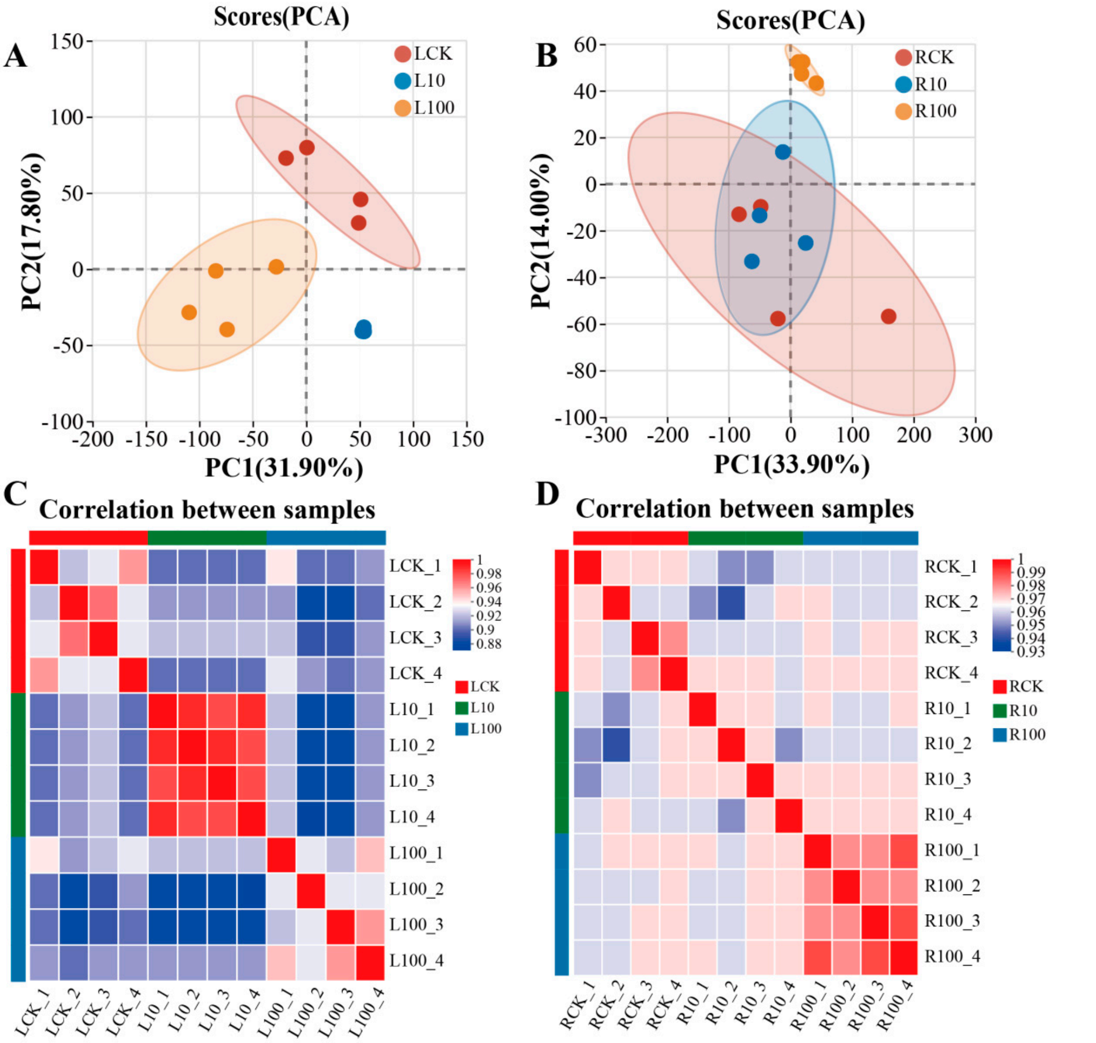

Figure S3. PCA analysis and Pearson correlation heatmap of metabolomic samples. (A) PCA of leaf samples (Ellipses show 95% confidence intervals for each group); (B) PCA of root samples (Ellipses show 95% confidence intervals for each group); (C) Pearson correlation analysis of leaf samples; (D) Pearson correlation analysis of root samples.
